# Window-Based Constant Beamwidth Beamformer

**DOI:** 10.3390/s19092091

**Published:** 2019-05-06

**Authors:** Tao Long, Israel Cohen, Baruch Berdugo, Yan Yang, Jingdong Chen

**Affiliations:** 1 Center of Intelligent Acoustics and Immersive Communications, Northwestern Polytechnical University, 127 Youyi West Road, Xi’an 710072, Shaanxi, China; y.yang@nwpu.edu.cn (Y.Y.); jingdongchen@ieee.org (J.C.); 2 Department of Electrical Engineering, Technion—Israel Institute of Technology, Technion City, Haifa 32000, Israel; icohen@ee.technion.ac.il (I.C.); bbaruch@technion.ac.il (B.B.)

**Keywords:** constant beamwidth beamformer, microphone arrays, chebyshev window, Kaiser window, discrete prolate spheroidal sequences

## Abstract

Beamformers have been widely used to enhance signals from a desired direction and suppress noise and interfering signals from other directions. Constant beamwidth beamformers enable a fixed beamwidth over a wide range of frequencies. Most of the existing approaches to design constant beamwidth beamformers are based on optimization algorithms with high computational complexity and are often sensitive to microphone mismatches. Other existing methods are based on adjusting the number of sensors according to the frequency, which simplify the design, but cannot control the sidelobe level. Here, we propose a window-based technique to attain the beamwidth constancy, in which different shapes of standard window functions are applied for different frequency bins as the real weighting coefficients of microphones. Thereby, not only do we keep the beamwidth constant, but we also control the sidelobe level. Simulation results show the advantages of our method compared with existing methods, including lower sidelobe level, higher directivity factor, and higher white noise gain.

## 1. Introduction

Beamformers, or spatial filters, enhance signals from a desired direction and suppress noise and interfering signals from other directions. Deterministic and adaptive beamforming techniques have been widely studied and used in radar, sonar, seismology, tomography, communication and many other areas [1,2,3,4,5].

A variety of beamforming techniques are available, including data-independent, statistically optimal and adaptive approaches. Traditional beamforming techniques suffer from a frequency varying beamwidth, which restricts their deployment in broad-band applications (e.g., speech communication). The basic approach of solving this problem is to design a constant beamwidth beamformer, where the beampattern maintains a fixed beamwidth over a wide frequency band. Many existing methods have been investigated to obtain constant beamwidths [6,7,8,9,10,11,12]. These methods are mainly based on optimization algorithms with high computational design complexity, and they are often sensitive to microphone mismatches.

Recently, Rosen et al. [13] proposed finite impulse response (FIR) based beamformers [14] with constant beamwidths. The main idea behind this approach is to change the effective array aperture in each frequency bin to maintain the beamwidth constant over the desired frequency band. This method is characterized by low computational complexity, but cannot control the sidelobe level. If we regard the coefficients of the FIR filter as a window function, then the beamformer is analogous to the discrete Fourier transform of the window. It can be shown that the FIR filter proposed in [13] is a kind of modified rectangular window, and it inspires us to use other kinds of windows. As a result, some standard window functions whose shapes are controlled by a single parameter can be directly used to obtain different beamwidths and sidelobe levels, e.g., a discrete prolate spheroidal sequences (DPSS) window can maximize the relative beamforming power that is concentrated in an angular region, and a Chebyshev window minimizes the beamwidth for a given sidelobe level.

In this paper, we propose window-based beamformers with constant beamwidths. The main idea is to apply different shapes of windows for different frequency bins as real weighting coefficients of microphones, so that the beamwidth is maintained constant by controlling the window parameters. The rest of this paper is organized as follows. In Section 2, we introduce the signal model and illustrate the frequency varying beamwidth problem for traditional uniform linear arrays. Section 3 proposes the window-based beamformer with a constant beamwidth, using modified rectangular, DPSS, Kaiser and Chebyshev windowss. Section 4 evaluates the performances of different window-based methods using the white noise gain and directivity factor. Finally, some conclusions are drawn in Section 5.

## 2. Signal Model and Problem Formulation

### 2.1. Signal Model

**Figure 1:**
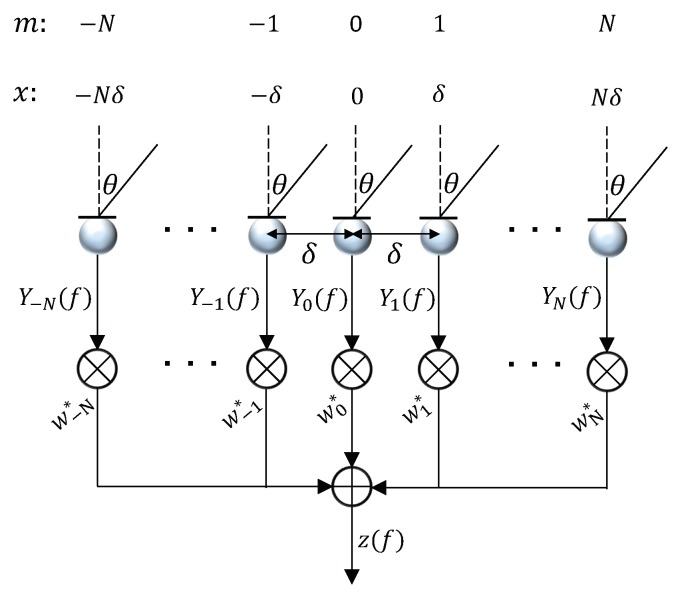
Beamforming with a uniform linear array consisting of *M* (M=2N+1) microphones with spacing δ, where *m* denotes the microphone index, *x* denotes the location of the microphone, θ is the direction of arrival angle measured with respect to the broadside of the linear array.

Consider a uniform linear array (ULA) consisting of *M* omnidirectional microphones, with an interelement spacing equal to δ. Assume that there are an odd number (M=2N+1) of microphones, as shown in Figure 1, whose locations are:

(1) $$\begin{array}{c} x_{m}=m\delta ,\;m=-N,-\left(N-1\right),\ldots ,N-1,N, \end{array}$$

where *m* denotes the microphone index, and $x_{m}$ denotes the location of the *m*th microphone.

We consider the farfield case where a source of interest radiates an acoustic wave that propagates in an anechoic environment at the speed of sound, i.e., c=340m/s. The direction of the source signal is parameterized by the angle θ measured with respect to the broadside of the linear array. In the frequency domain, the signal model at the frequency index *f* can be written as:

(2) $$\begin{array}{cc} Y_{m}\left(f\right) & =X_{m}\left(f\right)+V_{m}\left(f\right)\\ & =e^{-jm\left(2\pi f\delta /c\right)\sin\theta }X_{0}\left(f\right)+V_{m}\left(f\right), \end{array}$$

where $Y_{m}\left(f\right)$, $X_{m}\left(f\right)$, and $V_{m}\left(f\right)$ are the frequency-domain representations of the received noisy signal, the desired source signal and the additive noise signal at the *m*th microphone, respectively, f>0 is the temporal frequency, and *j* is the imaginary unit with $j^{2}=-1$. In a vector form, we can rearrange the signal model in Equation (2) as

(3) $$\begin{array}{cc} y\left(f\right) & =x\left(f\right)+v\left(f\right)\\ & =d\left(f,\theta \right)X_{0}\left(f\right)+v\left(f\right), \end{array}$$

where

(4) $$\begin{array}{cc} y\left(f\right) & \overset{▵}{=}{\left[\begin{array}{ccccc} Y_{-N}\left(f\right) & \cdots & Y_{0}\left(f\right) & \cdots & Y_{N}\left(f\right) \end{array}\right]}^{T},\\ x\left(f\right) & \overset{▵}{=}{\left[\begin{array}{ccccc} X_{-N}\left(f\right) & \cdots & X_{0}\left(f\right) & \cdots & X_{N}\left(f\right) \end{array}\right]}^{T},\\ v\left(f\right) & \overset{▵}{=}{\left[\begin{array}{ccccc} V_{-N}\left(f\right) & \cdots & V_{0}\left(f\right) & \cdots & V_{N}\left(f\right) \end{array}\right]}^{T}, \end{array}$$

the superscript ${}^{T}$ is the transpose operator, and

$$\begin{array}{c} d\left(f,\theta \right)\overset{▵}{=}{\left[\begin{array}{ccccc} e^{jN\left(2\pi f\delta /c\right)\sin\theta } & \cdots & 1 & \cdots & e^{-jN\left(2\pi f\delta /c\right)\sin\theta } \end{array}\right]}^{T}, \end{array}$$

is the signal propagation vector corresponding to θ, which is in the same form as the steering vector.

### 2.2. Beamformer

As shown in Figure 1, the beamformer estimates the desired signal by applying a spatial filter to the sensors’ outputs [2], i.e.,

(5) $$\begin{array}{cc} Z\left(f\right) & =\sum_{m=-N}^{N}w_{m}^{*}\left(f\right)Y_{m}\left(f\right)=w^{H}\left(f\right)y\left(f\right), \end{array}$$

where ${}^{*}$ and ${}^{H}$ denote complex conjugation and conjugate-transpose operator, $Z\left(f\right)$ is an estimate of the desired signal $X_{0}\left(f\right)$, and

(6) $$\begin{array}{c} w\left(f\right)\overset{▵}{=}{\left[\begin{array}{ccccc} w_{-N}\left(f\right) & \cdots & w_{0}\left(f\right) & \cdots & w_{N}\left(f\right) \end{array}\right]}^{T} \end{array}$$

is the linear filter of length 2N+1.

One of the most important measures to quantify the performance of a beamformer is the so-called beampattern or directivity pattern, which describes the sensitivity of the beamformer to a plane wave impinging on the array from the direction θ. Mathematically, the beampattern is defined as

(7) $$\begin{array}{cc} B\left(f,\theta \right) & \overset{▵}{=}w^{H}\left(f\right)d\left(f,\theta \right) \end{array}$$

(8) $$\begin{array}{c} =\sum_{m=-N}^{N}w_{m}^{*}\left(f\right)e^{-jm\left(2\pi f\delta /c\right)\sin\theta }. \end{array}$$

### 2.3. Beamwidth

We now give an example of the uniform weighting beamformer (i.e., delay-and-sum beamformer),

(9) $$\begin{array}{c} w_{m}=\frac{1}{M},\;m=-N,-\left(N-1\right),\ldots ,N-1,N, \end{array}$$

where M=2N+1. When θ≠0, we easily get

(10) $$\begin{array}{c} \left|B\left(f,\theta \right)\right|=\frac{1}{M}\left|\frac{1-e^{-j\left(2\pi Mf\delta /c\right)\sin\theta }}{1-e^{-j\left(2\pi f\delta /c\right)\sin\theta }}\right|. \end{array}$$

If we define the beamwidth $\theta_{\mathrm{BW}}$ as the angle between the two lowest values at both sides of the main lobe (i.e., the beamwidth null to null), $\theta_{\mathrm{BW}}$ can be obtained in this case:

(11) $$\begin{array}{cc} \theta_{\mathrm{BW}} & =2\sin^{-1}\left(\frac{c}{\delta Mf}\right). \end{array}$$

**Figure 2:**
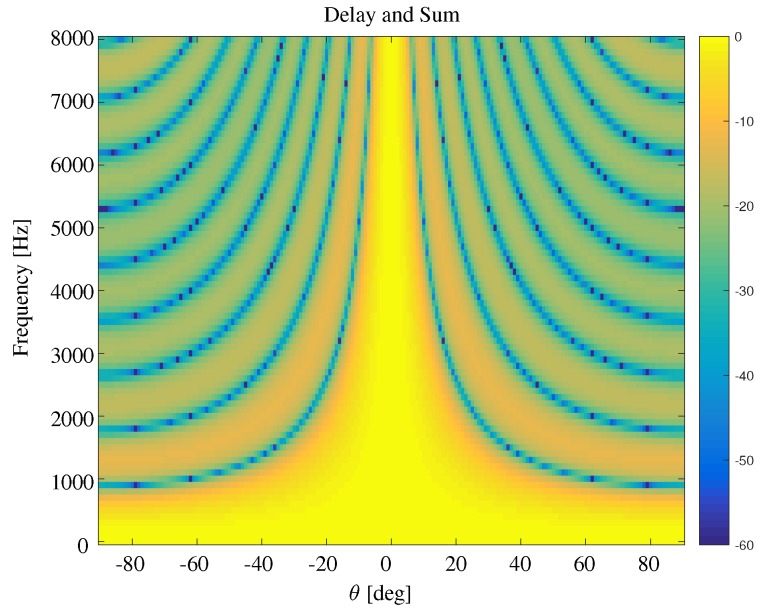
Beampattern using delay-and-sum beamformer. M=11, δ=3.5 cm.

This expression indicates the dependance of the beamwidth on the number of sensors *M*, interelement spacing δ and frequency *f*. One can observe that the beamwidth decreases as the frequency increases, which implies that this beamforming method suffers from a frequency varying beamwidth. The beampattern based on the delay-and-sum beamformer is shown in Figure 2. One observes that the beamwidth decreases as the frequency increases.

## 3. Window-Based Acoustic Beamformer with a Constant Beamwidth

In this section, we propose a window-based symmetrical beamformer method with a constant beamwidth $\theta_{\mathrm{CBW}}$ over a wide frequency range.

Define $u\overset{▵}{=}\left(2\pi f\delta /c\right)\sin\theta$, then the steering vector can be rewritten as:

(12) $$\begin{array}{c} d\left(u\right)={\left[\begin{array}{ccccc} e^{jNu} & \cdots & 1 & \cdots & e^{-jNu} \end{array}\right]}^{T}. \end{array}$$

Accordingly, the beampattern is obtained through the discrete Fourier transform of the spatial filter:

(13) $$\begin{array}{c} B\left(u\right)=\sum_{m=-N}^{N}w_{m}^{*}e^{-jmu}=w^{H}d\left(u\right) \end{array}$$

In this work, we restrict ourselves to real weights, then the beampattern is given by

(14) $$\begin{array}{c} B\left(u\right)=\sum_{m=-N}^{N}w_{m}e^{-jmu}=w^{T}d\left(u\right) \end{array}$$

The real weights $w_{m}$ are regarded as a spatial window function. Hence, the beamwidth can be maintained by applying different shapes of windows for different frequency bins. Next, we present four different kinds of windows, i.e., modified rectangular, DPSS, Kaiser, Chebyshev. For each window, we first introduce the mathematical representation, and then show how to control its shape as a function of frequency by setting the proper parameter of the window in order to maintain constant beamwidth.

### 3.1. Modified Rectangular Window

From Equation (11), in order to keep the beamwidth constant for varying frequency, the product Mf should remain constant, which means that the number of microphones should decrease as the frequency increases. To alleviate the beamwidth fluctuations, Rosen et al. [13] proposed a modified rectangular window based on smoothing coefficients.

The weights $w_{m}$ in [13] can be regarded as a kind of modified rectangular window:

(15) $$w_{m}=\left\{\begin{array}{cc} 1, & -K<m<K\\ g, & m=-K,m=K,(0<g<1)\\ 0, & \mathrm{otherwise} \end{array}\right.$$

where 2K+1 is the number of activated microphones, and *g* is the smoothing coefficient.

Next, we show how to obtain the parameter *K* and *g* to keep the beamwidth $\theta_{\mathrm{BW}}$ constant.

#### 3.1.1. Lowest and Highest Frequencies

Since the number of activated microphones should be less than *M* and more than 3, we should first determine the lowest frequency $f_{L}$ and the highest frequency $f_{H}$ for which the desired beamwidth is feasible. For the given array configuration and fixed beamwidth $\theta_{\mathrm{CBW}}$, based on Equation (11), the lowest and highest frequencies using rectangular window are

(16) $$\begin{array}{cc} f_{L,\mathrm{Rec}} & =\frac{c}{M\delta \sin\left(\theta_{\mathrm{CBW}}/2\right)},\\ f_{H,\mathrm{Rec}} & =\frac{c}{3\delta \sin\left(\theta_{\mathrm{CBW}}/2\right)}. \end{array}$$

Meanwhile, in order to avoid maximum grating lobe, the highest frequency $f_{H,\mathrm{Rec}}$ should also be smaller than c/δ.

#### 3.1.2. The Parameter *K*

For the feasible frequency range $f_{L}<f<f_{H}$, we need to reduce the number of activated microphones to keep (2K+1)f constant as the frequency varies. So the value of *K* is obtained by the maximum integer which satisfied $\left(2K+1\right)f⩽Mf_{L}$.

#### 3.1.3. The Parameter *g*

The smoothing coefficient *g* can be derived as follows. The beampattern using the modified rectangular window $w_{m}$ is given by

(17) $$\begin{array}{c} B\left(f,\theta \right)=\sum_{m=-N}^{N}w_{m}e^{-jm\left(2\pi f\delta /c\right)\sin\theta }\\ =2g\cos\left(2K\pi f\delta \sin\theta /c\right)+\sum_{m=-(K-1)}^{K-1}e^{-jm\left(2\pi f\delta /c\right)\sin\theta }. \end{array}$$

The optimal value of smoothing coefficient *g* is obtained by setting $B\left(f,\theta_{\mathrm{CBW}}/2\right)=0$, which yields

(18) $$\begin{array}{c} g=\frac{\sum_{m=-(K-1)}^{K-1}e^{-jm\left(2\pi f\delta /c\right)\sin\left(\theta_{\mathrm{CBW}}/2\right)}}{-2\cos\left(2K\pi f\delta \sin\left(\theta_{\mathrm{CBW}}/2\right)/c\right)}. \end{array}$$

In order to normalize the beampattern, we use the normalized weighting coefficients as:

(19) $$\begin{array}{c} w_{m}^{{}^{\prime }}=\frac{w_{m}}{\sum_{m=-N}^{N}w_{m}}=\frac{w_{m}}{2g+2K+1}. \end{array}$$

**Figure 3:**
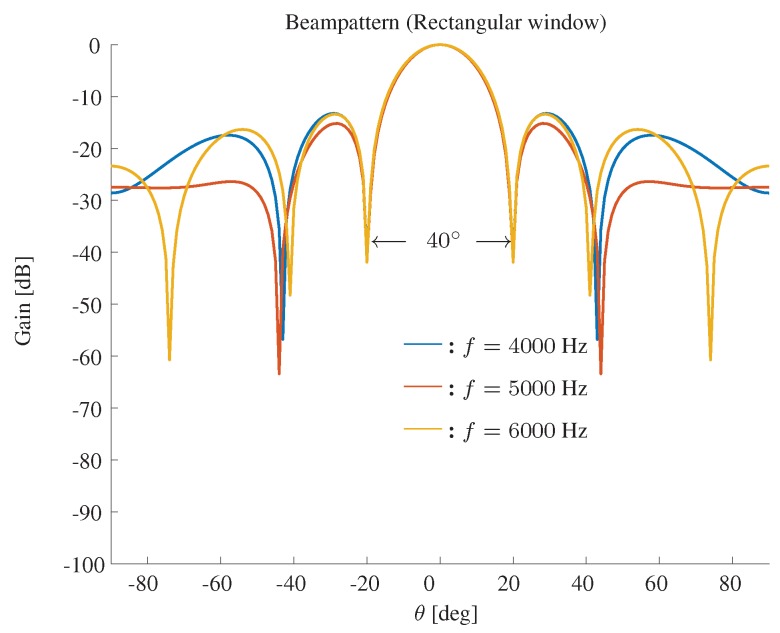
Constant beamwidth beampattern using a modified rectangular window. The beamwidth is fixed to $40^{\circ }$, M=11, δ=3.5 cm.

The beampattern based on a modified rectangular window is shown in Figure 3. The beamwidth is fixed to $40^{\circ }$, M=11 and δ=3.5 cm. We show the beampattern in three different frequencies f=4000,5000,6000 Hz. One observes that the rectangular window-based method can effectively fix the beamwidth, but the sidelobe level is high in this case.

### 3.2. DPSS Window

Rectangular window-based method can effectively fix the beamwidth but cannot control the sidelobes. So, we attempt to find some other windows with real weights in order to control the sidelobe level. Based on Equation (13), we can first define α as the ratio of the total beamforming power that is concentrated in a given angular region:

(20) $$\begin{array}{cc} \alpha & =\frac{\int_{-u_{0}}^{u_{0}}{\left|B(u)\right|}^{2}du}{\int_{-\pi }^{\pi }{\left|B(u)\right|}^{2}du}\\ & =\frac{w^{H}\left[\int_{-u_{0}}^{u_{0}}d\left(u\right)d{\left(u\right)}^{H}du\right]w}{w^{H}\left[\int_{-\pi }^{\pi }d\left(u\right)d{\left(u\right)}^{H}du\right]w}\\ & =\frac{w^{H}Aw}{w^{H}Bw}, \end{array}$$

where $u_{0}=\left(2\pi f\delta /c\right)\sin\theta_{0}$, $A=\int_{-u_{0}}^{u_{0}}d\left(u\right)d{\left(u\right)}^{H}du$, the (m,n)th element of A is

(21) $$\begin{array}{c} \int_{-u_{0}}^{u_{0}}e^{jmu}e^{-jnu}du=\frac{2\sin\left[\left(m-n\right)u_{0}\right]}{m-n}, \end{array}$$

and similarly,

(22) $$\begin{array}{c} B=\int_{-\pi }^{\pi }d\left(u\right)d{\left(u\right)}^{H}du=2\pi I, \end{array}$$

where I is the M×M identity matrix. To maximize α, the optimum solution is obtained from the eigenvalue problem

(23) $$\begin{array}{c} Aw=\pi \lambda w \end{array}$$

or equivalently,

(24) $$\begin{array}{c} \sum_{n=-N}^{N}\frac{\sin\left[\left(m-n\right)u_{0}\right]}{m-n}w_{n}=\pi \lambda w_{m}, \end{array}$$

where λ is an eigenvalue of A. Thus in this case, α is maximized by the maximum eigenvalue $\lambda_{\max}$. The resulting weight sequences $w_{m}$ are called discrete prolate spheroidal sequences [15] (DPSS, or Slepian sequences).

#### 3.2.1. Lowest and Highest Frequencies

When $u_{0}=0$, the DPSS window becomes a rectangular window. From Equation (11), we get the lowest frequency $f_{L,\mathrm{DPSS}}$ which enables the desired beamwidth:

(25) $$\begin{array}{cc} f_{L,\mathrm{DPSS}} & =\frac{c}{M\delta \sin\left(\theta_{\mathrm{CBW}}/2\right)}, \end{array}$$

On the other side, in order to avoid maximum grating lobe, the highest frequency is given by

(26) $$\begin{array}{cc} f_{H,\mathrm{DPSS}} & =\frac{c}{\delta }. \end{array}$$

#### 3.2.2. The Parameter $u_{0}$

For the given array configuration, it is easy to verify that the beamwidth increases as the parameter $u_{0}$ increases. For the given constant beamwidth $\theta_{\mathrm{CBW}}$, we can set for the frequency *f*

$$u_{0}\approx \left(2\pi f\delta /c\right)\sin\left(\theta_{\mathrm{CBW}}/2\right),$$

or we can search the optimal parameter $u_{0}$ to fix the beamwidth in practice, then $w_{m}$ can be obtained following the DPSS window in Equation (24).

At last, in order to normalize the beampattern, we use the normalized weighting coefficients as:

(27) $$\begin{array}{c} w_{m}^{{}^{\prime }}=\frac{w_{m}}{\sum_{m=-N}^{N}w_{m}}. \end{array}$$

**Figure 4:**
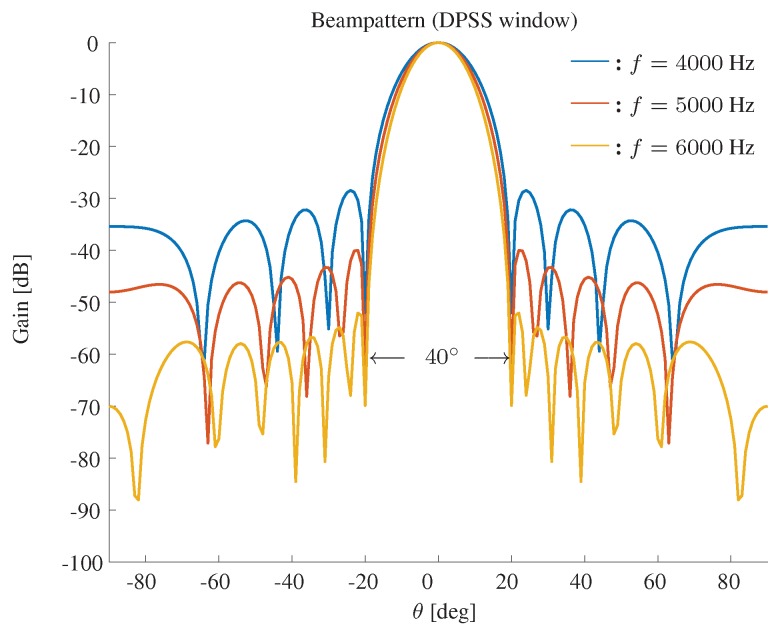
Constant beamwidth beampattern using a DPSS window. The beamwidth is fixed to $40^{\circ }$, M=11, δ=3.5 cm.

The beampattern based on a DPSS window is shown in Figure 4. The beamwidth is still fixed to $40^{\circ }$, M=11 and δ=3.5 cm. We show the beampattern in three different frequencies f=4000,5000,6000 Hz. It can be seen that the DPSS window based approach cannot only keep the beamwidth constant, but also effectively suppresses the sidelobe level.

### 3.3. Kaiser Window

A Kaiser window is a simple approximation to the DPSS window using Bessel functions. The details of calculating a Kaiser window can be found in [16]. The weighting coefficient of the microphone with index *m* is:

(28) $$\begin{array}{cc} w_{m}=\frac{J_{0}\left(\beta \sqrt{1-{\left(\frac{m}{N}\right)}^{2}}\right)}{J_{0}\left(\beta \right)}, & -N⩽m⩽N \end{array}$$

where $J_{0}$ is the zeroth-order modified Bessel function of the first kind. The parameter β⩾0 specifies a beampattern tradeoff between the sidelobe amplitude $A_{\mathrm{SL}}$ and the main lobe width. When β=0, the Kaiser window becomes a rectangular window.

#### 3.3.1. Lowest and Highest Frequencies

Since the Kaiser window approximates the DPSS window, in oder to make the desired beamwidth feasible, the lowest and highest frequencies using kaiser window are

(29) $$\begin{array}{cc} f_{L,\mathrm{Kaiser}} & =\frac{c}{M\delta \sin\left(\theta_{\mathrm{CBW}}/2\right)},\\ f_{H,\mathrm{Kaiser}} & =\frac{c}{\delta }. \end{array}$$

#### 3.3.2. The Parameter β

For the given array configuration, it is easy to verify that the beamwidth increases as the parameter β increases. For a given constant beamwidth $\theta_{\mathrm{CBW}}$, the parameter β can be obtained following the approximate piecewise relation [16]:

$$\begin{array}{c} \left\{\begin{array}{c} \beta \approx 0.76608{\left(A_{\mathrm{SL}}-13.26\right)}^{0.4}+0.09834\left(A_{\mathrm{SL}}-13.26\right),\\ A_{\mathrm{SL}}\approx \left(26Mf\delta /c\right)\sin\left(\theta_{\mathrm{CBW}}/2\right)-12. \end{array}\right. \end{array}$$

In practice, we can also search the optimal parameter β for a constant beamwidth. Furthermore, in order to normalize the beampattern, we use the normalized weighting coefficients as:

(30) $$\begin{array}{c} w_{m}^{{}^{\prime }}=\frac{w_{m}}{\sum_{m=-N}^{N}w_{m}}. \end{array}$$

**Figure 5:**
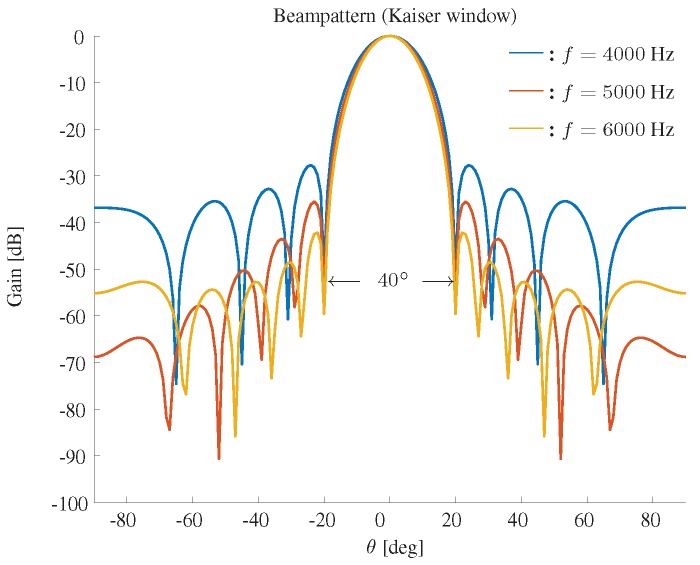
Constant beamwidth beampattern using a Kaiser window. The beamwidth is fixed to $40^{\circ }$, M=11, δ=3.5 cm.

The beampattern based on a Kaiser window is shown in Figure 5. We fix the beamwidth to $40^{\circ }$, M=11 and δ=3.5 cm, and plot the beampattern in three different frequencies f=4000,5000,6000 Hz. It is shown that the Kaiser window based method can get a similar beampattern as the DPSS window.

### 3.4. Chebyshev Window

Another window that can be used to control the main lobe beamwidth and sidelobe level is the Chebyshev Window [17,18], which minimizes the beamwidth for a given maximum sidelobe level. The coefficients $w_{m}$ of the Chebyshev window are given by

(31) $$\begin{array}{c} w_{m}=\frac{1}{M}\left[1+2r\sum_{n=1}^{N}T_{2N}\left(x_{0}\cos\left(\frac{n\pi }{M}\right)\right)\cos\left(\frac{2\pi nm}{M}\right)\right], \end{array}$$

where −N⩽m⩽N, M=2N+1, $x_{0}=\cosh\left(\frac{1}{2N}\cosh^{-1}\left(\frac{1}{r}\right)\right)$, *r* is defined as the amplitude ratio between maximum sidelobe and mainlobe, and $T_{m}\left(x\right)$ is the Chebyshev polynomial of the first kind, defined by

(32) $$\begin{array}{c} T_{m}\left(x\right)=\left\{\begin{array}{cc} \cos\left(m\cos^{-1}\left(x\right)\right) & |x|⩽1\\ \cosh\left(m\cosh^{-1}\left(x\right)\right) & |x|>1 \end{array}\right. \end{array}$$

#### 3.4.1. Lowest and Highest Frequencies

When the sidelobe attenuation is the same as the mainlobe, or r=1, the coefficients $w_{m}$ of the Chebyshev window are

(33) $$w_{m}=\left\{\begin{array}{cc} 1, & m=-N,m=N\\ 0, & \mathrm{otherwise}. \end{array}\right.$$

It is equivalent to a two elements array with an interelement spacing 2Nδ. According to Equations (25) and (26), the lowest and highest frequencies using Chebyshev window are:

(34) $$\begin{array}{cc} f_{L,\mathrm{Cheb}} & =\frac{c}{4N\delta \sin\left(\theta_{\mathrm{CBW}}/2\right)},\\ f_{H,\mathrm{Cheb}} & =\frac{c}{\delta }. \end{array}$$

#### 3.4.2. The Parameter *r*

For the given array configuration, it is easy to verify that the beamwidth increases as the parameter *r* decreases. For a given constant beamwidth $\theta_{\mathrm{CBW}}$, the parameter *r* can be obtained following the approximate piecewise relation:

$$\begin{array}{c} \left\{\begin{array}{cc} r & =1/\cosh\left(2N\cosh^{-1}x_{0}\right),\\ x_{0} & \approx 1/\cos\left(\left(\pi f\delta /c\right)\sin\left(\frac{\theta_{\mathrm{CBW}}}{2}\right)\right). \end{array}\right. \end{array}$$

In practice, we can also search the optimal parameter *r* for a constant beamwidth. Again, in order to normalize the beampattern, we use the normalized weighting coefficients as:

(35) $$\begin{array}{c} w_{m}^{{}^{\prime }}=\frac{w_{m}}{\sum_{m=-N}^{N}w_{m}}. \end{array}$$

**Figure 6:**
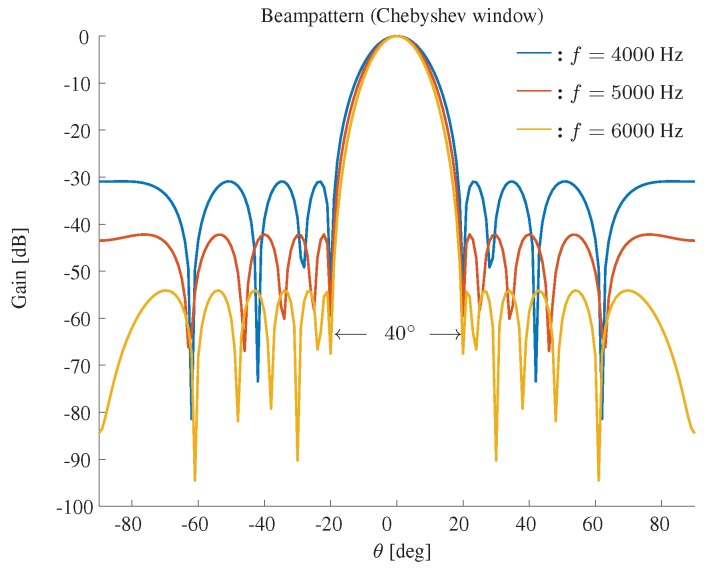
Constant beamwidth beampattern using the Chebyshev window. The beamwidth is fixed to $40^{\circ }$, M=11, δ=3.5 cm.

The beampatterns for different frequencies based on a Chebyshev window are shown in Figure 6. Again, the beamwidth is fixed to $40^{\circ }$, and one can find that the Chebyshev window based method can also effectively keep the beamwidth constant and yield equi-level sidelobes.

## 4. Experiments and Results

We have discussed different window-based beamformers with a constant beamwidth in Section 3. In this section, we compare the performances of different windows via several simulations. All of the simulated uniform linear arrays are configured with M=11 omnidirectional microphones, with an interelement spacing equal to δ=3.5. In these experiments, the modified rectangular, DPSS, Kaiser and Chebyshev window-based methods are used and the beamwidth is fixed to $\theta_{\mathrm{CBW}}=40^{\circ }$. In Section 4.1 we show how to set the optimal parameter to shape the window and design the beamformer in order to attain beamwidth constancy. Section 4.2 provides the performance measures of the beamformer. Section 4.3 shows the results.

### 4.1. Optimal Window Parameter

In Section 3, we have shown how to set the parameter to shape the window in order to attain beamwidth constancy, e.g., the parameter *g* for the modified rectangular window, the parameter $u_{0}$ for the DPSS window, the parameter β for the Kaiser window, and the parameter *r* for the Chebyshev window. Compared with the optimization-based method, one can see that our method has very low computational complexity in the design process, because the problem has been simplified as obtaining a single parameter of the standard window.

In practice, we can also search the optimal parameter of a given window. There are two reasons for using the search method: (1) The relationship between the fixed beamwidth and the parameter is approximate piecewise in some cases. (2) The search method can be easily extended to non uniform arrays. Algorithm 1 shows the search algorithm of a window-based beamformer with a constant beamwidth, where we search the optimal parameter of a given window for each frequency bin to keep the beamwidth fixed to $\theta_{\mathrm{CBW}}=40^{\circ }$, $f_{L}$ and $f_{H}$ are the lower and upper cutoff frequencies of the frequency band, respectively.

**Algorithm 1** Algorithm for Searching the Optimal Parameter.**for**$f=f_{L}:f_{H}$ Initial the parameter of window w Search $\theta_{\min}$ for the first lowest value of $\left|B\left(f,\theta \right)\right|=\left|w^{H}\left(f\right)d\left(f,\theta \right)\right|$ **while**
$\theta_{\min}\neq \theta_{\mathrm{CBW}}/2$  Increase (or decrease) the parameter of window w  Search $\theta_{\min}$ for the first lowest value of $\left|B\left(f,\theta \right)\right|=\left|w^{H}\left(f\right)d\left(f,\theta \right)\right|$ **end** **end**

### 4.2. Performance Measures

We evaluate the beamformers using white noise gain (WNG) and directivity factor (DF) [1,4,19]. The WNG is a measure indicating the array gain in the presence of uncorrelated white noise, which is also a measure of the sensitivity of the microphone array to some of its imperfections, such as sensor noise and mismatch. The DF of the array is the gain in signal-to-noise ratio (SNR) for the case of spherical diffuse noise. Mathematically, they are respectively defined as W and D (note that the main lobe is perpendicular to the line that connects all the array elements):

(36) $$\begin{array}{c} W\left[w\left(f\right)\right]=\frac{{\left|w^{H}\left(f\right)d\left(f,0\right)\right|}^{2}}{w^{H}\left(f\right)w\left(f\right)}, \end{array}$$

(37) $$\begin{array}{cc} D\left[w\left(f\right)\right] & =\frac{{\left|B\left[w\left(f\right),0\right]\right|}^{2}}{\frac{1}{2}\int_{-\frac{\pi }{2}}^{\frac{\pi }{2}}{\left|B\left[w\left(f\right),\theta \right]\right|}^{2}\sin\theta d\theta }\\ & =\frac{{\left|w^{H}\left(f\right)d\left(f,0\right)\right|}^{2}}{\frac{1}{2}\int_{-\frac{\pi }{2}}^{\frac{\pi }{2}}w^{H}\left(f\right)d\left(f,\theta \right)d{\left(f,\theta \right)}^{H}w\left(f\right)\sin\theta d\theta }. \end{array}$$

We also evaluate the beamformers beamwidth which is defined as the angle between the two lowest values at both sides of the main lobe (i.e., the beamwidth null to null).

### 4.3. Results

#### 4.3.1. Wideband Beampatterns with a Constant Beamwidth Using Different Windows

**Figure 7:**
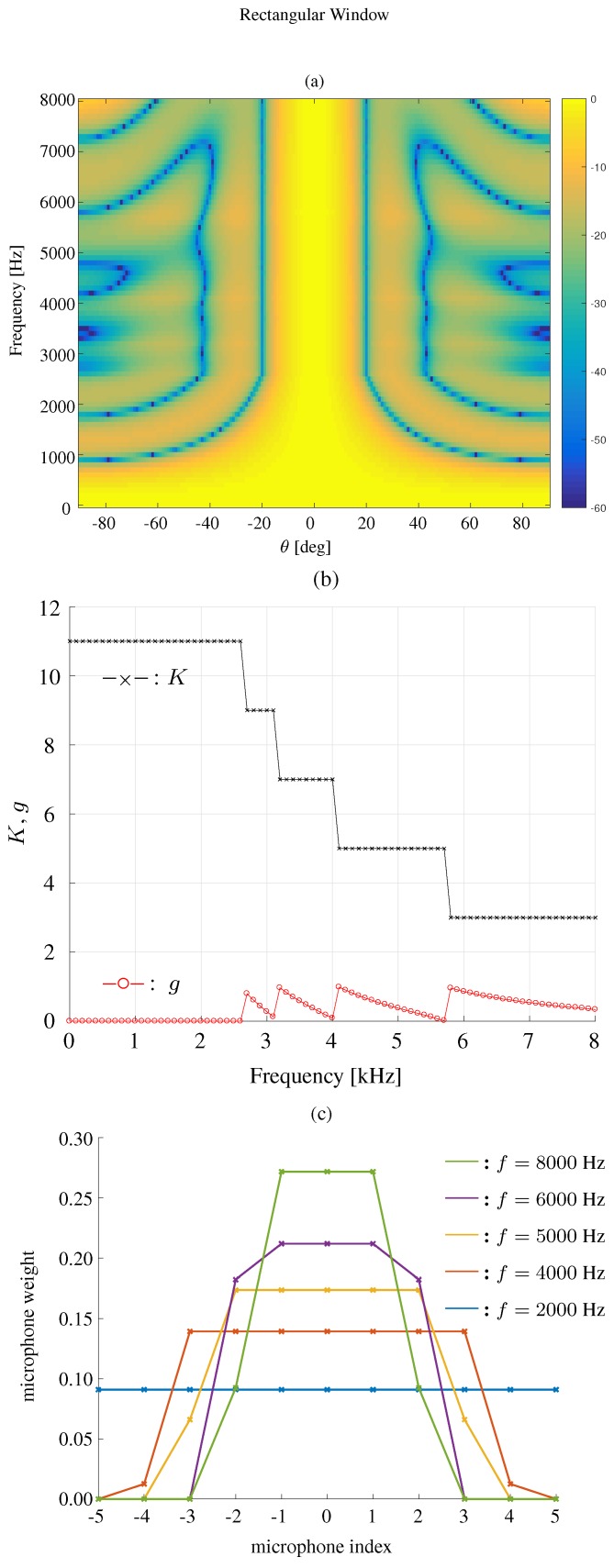
Constant beamwidth beamforming using the modified rectangular window-based method: (**a**) constant beamwidth beampattern, (**b**) the parameter K,g, (**c**) the weights of microphones for different frequency bins. The beamwidth is fixed to $40^{\circ }$, M=11, δ=3.5 cm.

**Figure 8:**
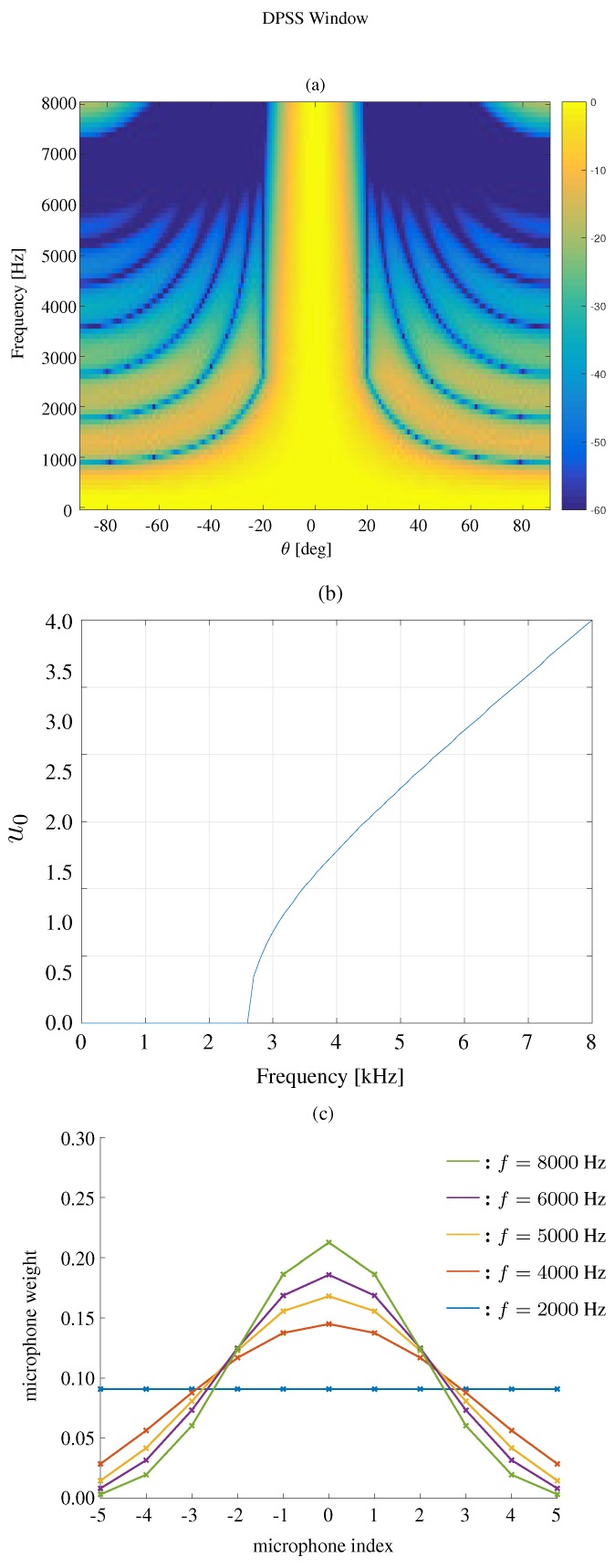
Constant beamwidth beamforming using the DPSS window-based method: (**a**) constant beamwidth beampattern, (**b**) the parameter $u_{0}$, (**c**) the weights of microphones for different frequency bins. The beamwidth is fixed to $40^{\circ }$, M=11, δ=3.5 cm.

**Figure 9:**
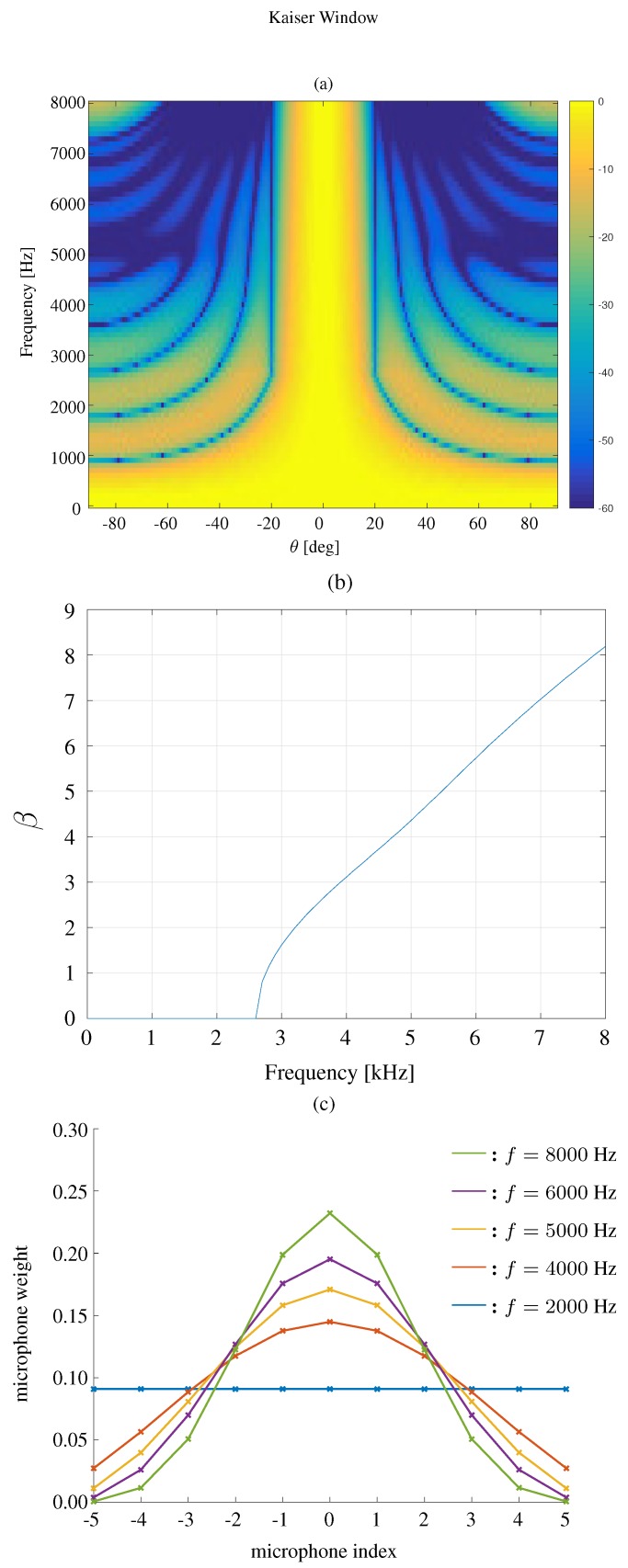
Constant beamwidth beamforming using the Kaiser window-based method: (**a**) constant beamwidth beampattern, (**b**) the parameter β, (**c**) the weights of microphones for different frequency bins. The beamwidth is fixed to $40^{\circ }$, M=11, δ=3.5 cm.

**Figure 10:**
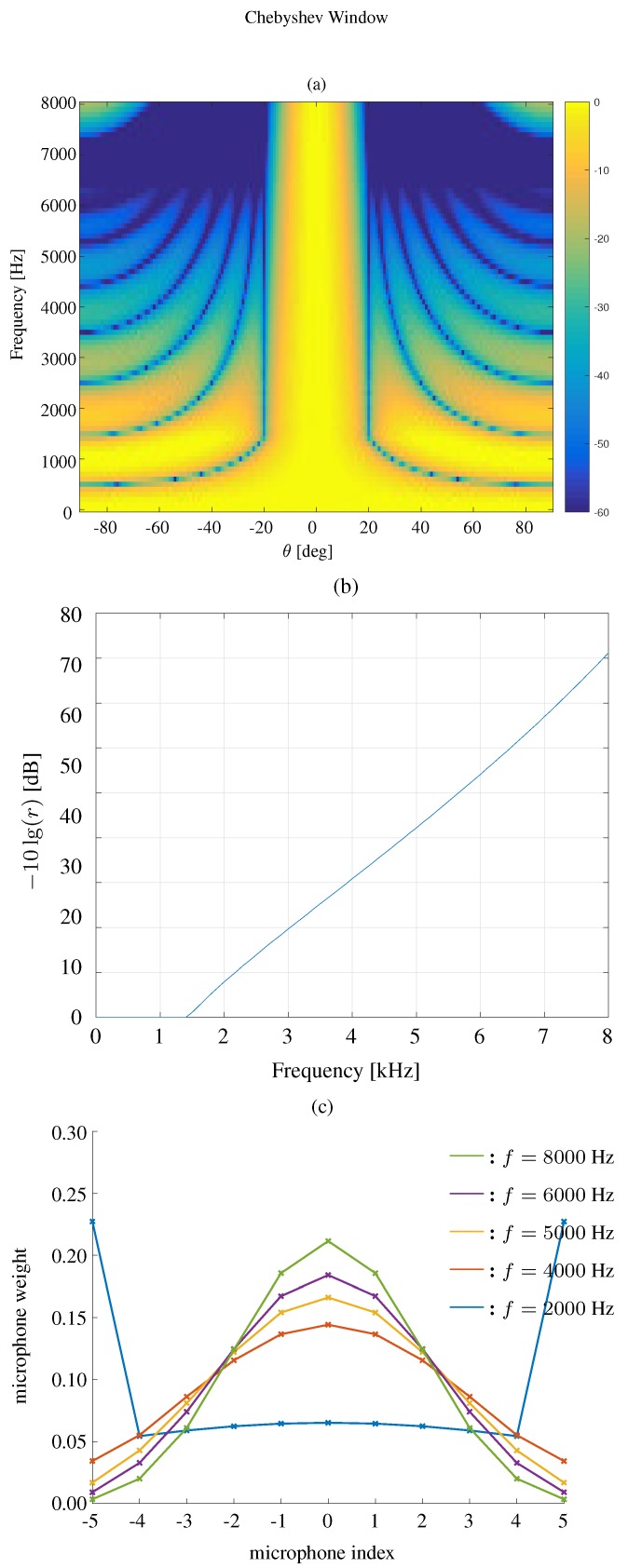
Constant beamwidth beamforming using the Chebyshev window-based method: (**a**) constant beamwidth beampattern, (**b**) the parameter *r*, (**c**) the weights of microphones for different frequency bins. The beamwidth is fixed to $40^{\circ }$, M=11, δ=3.5 cm.

We first compare the constant beamwidth beampatterns using different kinds of windows, where the frequency range is 0<f<8000 Hz. The results are plotted in Figure 7 for the modified rectangular window, Figure 8 for the DPSS window, Figure 9 for the Kaiser window, and Figure 10 for the Chebyshev window. One can see that all the window-based methods can effectively keep the beamwidth constant over a wide frequency band. Compared with modified rectangular window [13], the other proposed windows can obtain much lower sidelobe levels.

#### 4.3.2. Chebyshev Window

**Figure 11:**
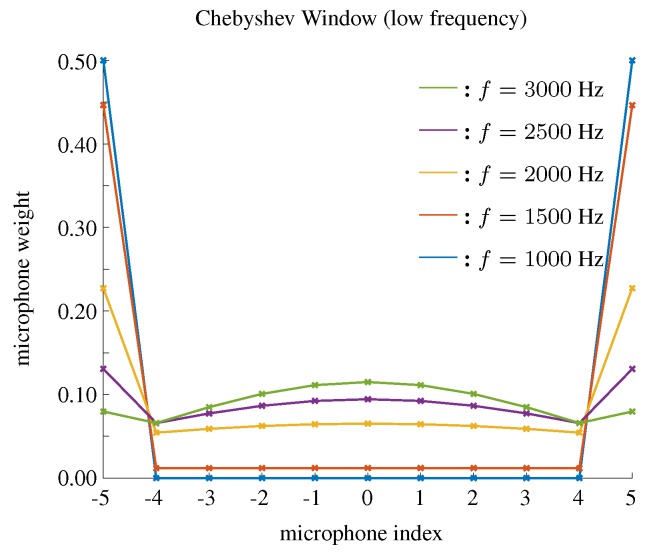
The weights of microphones for low frequency bins using the Chebyshev window, M=11, δ=3.5 cm.

In order to explain the performance based on the Chebyshev window in low frequency bins, we show the weights of the microphones using the Chebyshev window when *f* = 1000, 1500, 2000, 2500, 3000 Hz, and the results are plotted in Figure 11. One can find that the Chebyshev approach designs a kind of ’saddle’ shape window, where high weights are set for the microphones at the edges of the array. Suppose an extreme situation that we only use the two microphones at the edge, which means the Chebyshev window will be

(38) $$\begin{array}{c} w={\left[\begin{array}{ccccccc} 0.5 & 0 & \cdots & 0 & \cdots & 0 & 0.5 \end{array}\right]}^{T}, \end{array}$$

the interelement spacing will be $\delta^{\prime }=2N\delta$ and the number of microphones is $M^{\prime }=2$. Based on Equation (11), the lowest frequency $f_{L,\mathrm{Cheb}}$ which can attain the fixed beamwidth $\theta_{\mathrm{CBW}}$ is

(39) $$\begin{array}{cc} f_{L,\mathrm{Cheb}} & =\frac{c}{M^{\prime }\delta^{\prime }\sin\left(\theta_{\mathrm{CBW}}/2\right)}=\frac{c}{4N\delta \sin\left(\theta_{\mathrm{CBW}}/2\right)}\\ & <\frac{c}{M\delta \sin\left(\theta_{\mathrm{CBW}}/2\right)}=f_{L,\mathrm{Rec}}=f_{L,\mathrm{DPSS}}=f_{L,\mathrm{Kaiser}}. \end{array}$$

With this method, it is equivalent to increasing the interelement spacing and the virtual length of the array. As a result, the Chebyshev window-based beamformer can attain beamwidth constancy in lower frequencies compared with the other windows-based beamformers.

**Figure 12:**
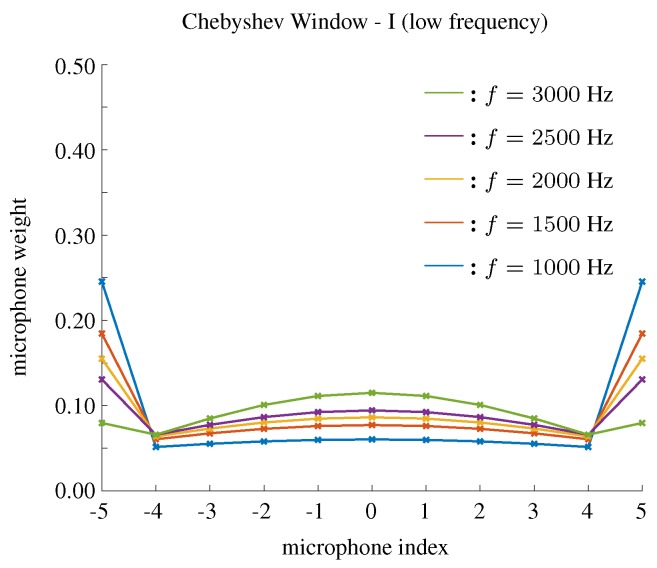
The weights of microphones for low frequency bins using the Chebyshev window-I, M=11, δ=3.5 cm.

**Figure 13:**
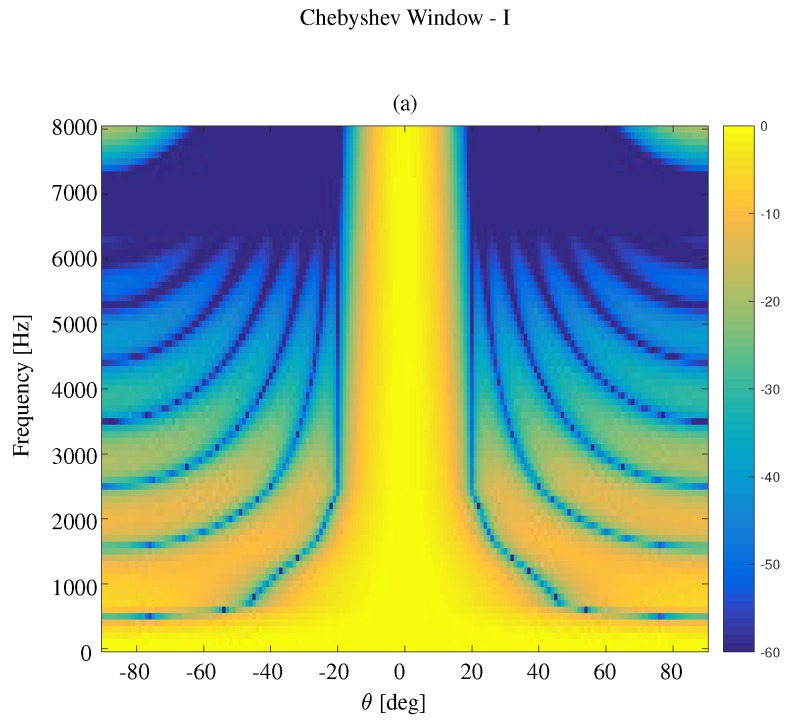

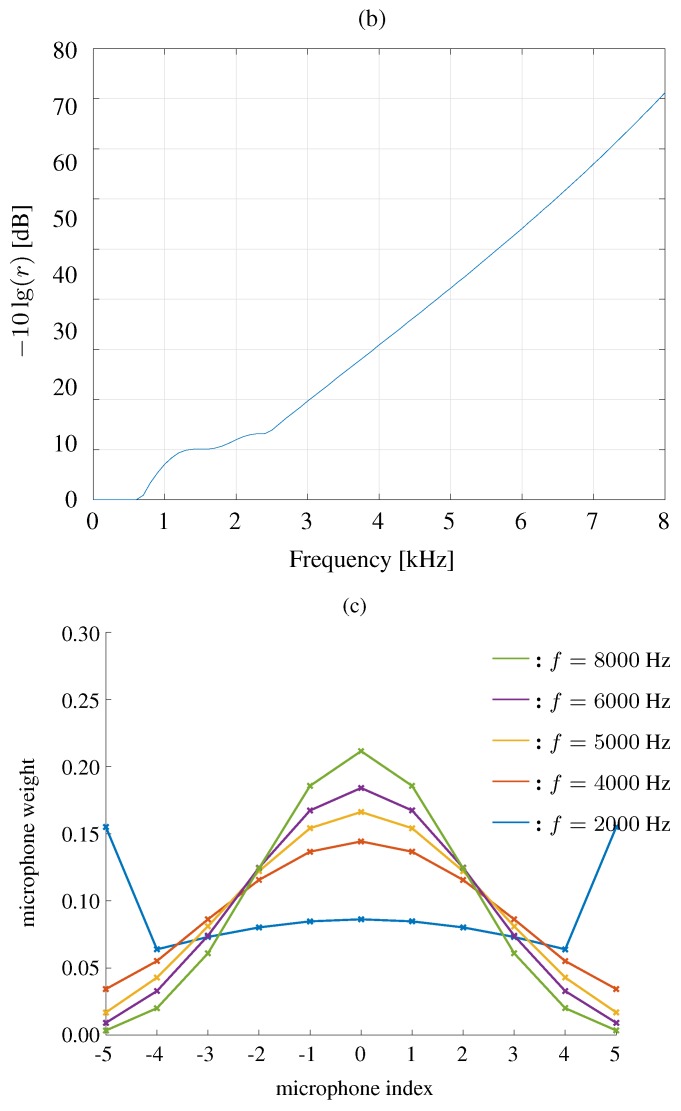
Constant beamwidth beamforming using the Chebyshev window-I method: (**a**) constant beamwidth beampattern, (**b**) the parameter *r*, (**c**) the weights of microphones for different frequency bins. The beamwidth is fixed to $40^{\circ }$, M=11, δ=3.5 cm.

In order to improve the DF using a Chebyshev window in low frequency bins, we can search the parameter *r* to reach a compromise between beamwidth and DF. We call this compromised method a Chebyshev window-I. The search criteria in this case will increase (or decrease) the parameter of the window to maximize the directivity index, given the beamwidth is above the desired minimal beamwidth. Figure 12 shows the weights of microphones using Chebyshev window-I when f=1000,1500,2000,2500,3000. The beampatterns and the parameters as functions of frequency using this method are shown in Figure 13.

#### 4.3.3. Directivity Factor and White Noise Gain as Function of Frequency

**Figure 14:**
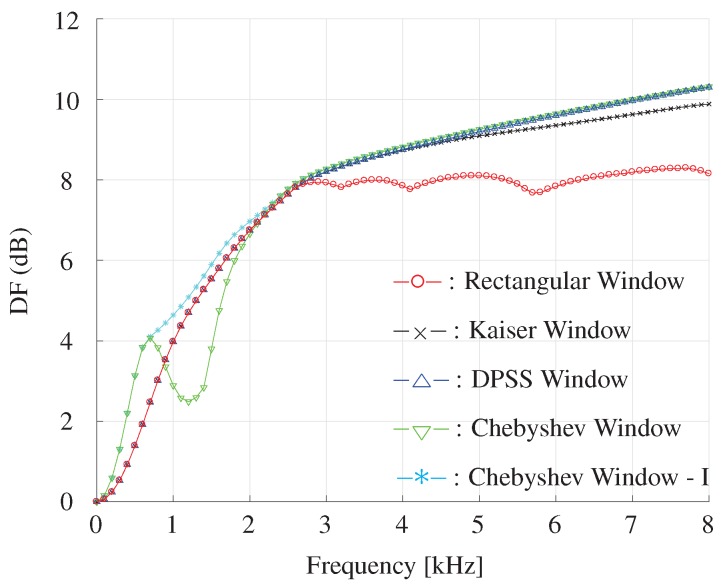
DF as a function of frequency for different window-based beamformers with a constant beamwidth. The beamwidth is fixed to $40^{\circ }$, M=11, δ=3.5 cm.

**Figure 15:**
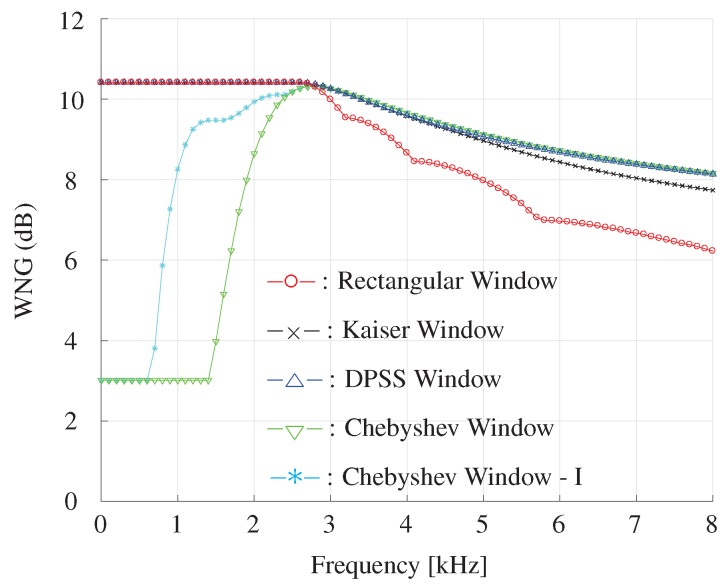
WNG as a function of frequency for different window-based beamformers with a constant beamwidth. The beamwidth is fixed to $40^{\circ }$, M=11, δ=3.5 cm.

The DF and WNG as functions of frequency for different window-based beamformer are plotted in Figure 14 and Figure 15. It is shown that the DPSS, Kaiser and Chebyshev window-based beamformer can achieve higher WNG and DF compared with the rectangular window in high frequencies. For frequencies below 2500 Hz, one can also see that the Chebyshev window-I beamformer gets highest DF compared with the other beamformers.

#### 4.3.4. Beamwidth as Function of Frequency

**Figure 16:**
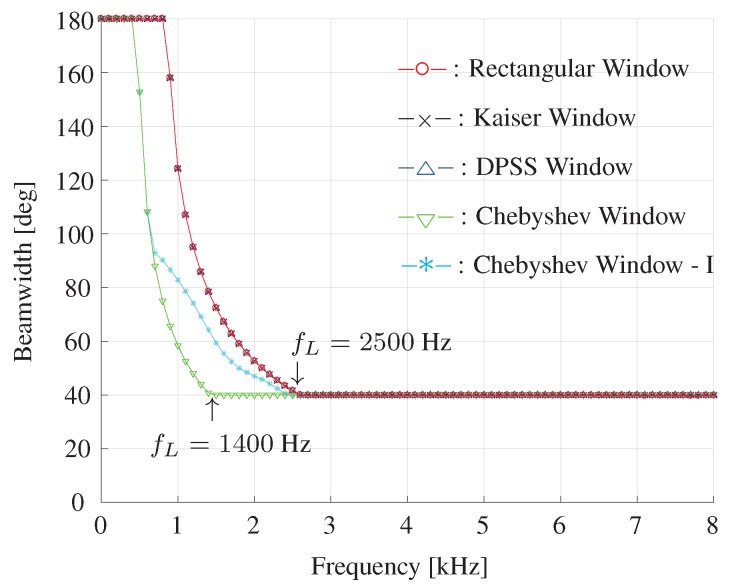
Beamwidth as a function of frequency for different window-based beamformers. The beamwidth is fixed to $40^{\circ }$, M=11, δ=3.5 cm.

At last, we compare the beamwidth as function of frequency for different window-based beamformers, and the results are plotted in Figure 16. For high frequencies (2500<f<8000 Hz), all the window-based beamformers can effectively fix the beamwidth to be $40^{\circ }$. For frequencies below 2500 Hz, it can be found that the Chebyshev window-based beamformer obtains smaller beamwidth compared with the other beamformers. As a result, the Chebyshev window-based beamformer can reach to the fixed beamwidth ($40^{\circ }$) at nearly 1400 Hz, but the other window-based beamformers attain beamwidth constancy only above 2500 Hz.

## 5. Conclusions

Traditional beamforming techniques suffer from a frequency varying beamwidth, which restricts their deployment in broadband applications. We have proposed window-based beamformers with constant beamwidths. Our method can effectively fix the beamwidth and exhibits the following advantages: (1) Compared with the optimization-based method, the proposed window-based approach is characterized by lower computational design complexity and higher white noise gain (which means it is less sensitive to microphone mismatches). (2) Compared with the FIR-based method proposed in [13], our window-based approach can reduce the sidelobe level and obtain higher directivity factor. Experiments corroborate the theoretical analysis and show that we can adjust the parameter of window to get the tradeoff between WNG and DF. Furthermore, hybrid window based beamformer design method is a topic for future research, which facilitates different windows for different frequency bins.

## References

[1] Benesty J., Cohen I., Chen J. (2018). Fundamentals of Signal Enhancement and Array Signal Processing.

[2] Benesty J., Chen J., Huang Y. (2008). Microphone Array Processing.

[3] Van Trees H.L. (2004). Optimum Array Processing: Part IV of Detection, Estimation, and Modulation Theory.

[4] Benesty J., Chen J., Cohen I. (2015). Design of Circular Differential Microphone Arrays.

[5] Darsena D., Verde F. (2007). Minimum-Mean-Output-Energy Blind Adaptive Channel Shortening for Multicarrier SIMO Transceivers. IEEE Trans. Signal Process..

[6] Ward D.B., Kennedy R.A., Williamson R.C. (1996). FIR filter design for frequency invariant beamformers. IEEE Signal Process. Lett..

[7] Parra L.C. (2006). Steerable frequency-invariant beamforming for arbitrary arrays. J. Acoust. Soc. Am..

[8] Liu W., Weiss S., McWhirter J.G., Proudler I.K. (2007). Frequency invariant beamforming for two-dimensional and three-dimensional arrays. Signal Process..

[9] Markovich S., Gannot S., Cohen I. (2009). Multichannel eigenspace beamforming in a reverberant noisy environment with multiple interfering speech signals. IEEE Trans. Audio Speech Lang. Process..

[10] Crocco M., Trucco A. (2011). Design of robust superdirective arrays with a tunable tradeoff between directivity and frequency-invariance. IEEE Trans. Signal Process..

[11] Tourbabin V., Agmon M., Rafaely B., Tabrikian J. (2012). Optimal real-weighted beamforming with application to linear and spherical arrays. IEEE Trans. Audio Speech Lang. Process..

[12] Wu Y.I., Wong K.T., Yuan X., Lau S.K., Tang S.K. (2012). A directionally tunable but frequency-invariant beamformer on an acoustic velocity-sensor triad to enhance speech perception. J. Acoust. Soc. Am..

[13] Rosen O., Cohen I., Malah D. (2017). FIR-based symmetrical acoustic beamformer with a constant beamwidth. Signal Process..

[14] Ward D.B., Williamson R.C., Kennedy R.A. (1998). Broadband microphone arrays for speech acquisition. Acoust. Aust..

[15] Slepian D., Pollak H.O. (1961). Prolate spheroidal wave functions, Fourier analysis and uncertainty. Bell Labs Tech. J..

[16] Kaiser J.F. Nonrecursive digital filter design using the I_0-sinh window function. Proceedings of the IEEE International Symposium on Circuits & Systems.

[17] Dolph C. (1946). A current distribution for broadside arrays which optimizes the relationship between beam width and side-lobe level. Proc. IRE.

[18] Lyons R.G. (2011). Understanding Digital Signal Processing.

[19] Pan C., Chen J., Benesty J. (2015). Theoretical analysis of differential microphone array beamforming and an improved solution. IEEE Trans. Audio Speech Lang. Process..

